# Minimally Invasive Esophagectomy for Esophageal Cancer: The First Experience from Pakistan

**DOI:** 10.1155/2014/864705

**Published:** 2014-07-20

**Authors:** Farrukh Hassan Rizvi, Syed Shahrukh Hassan Rizvi, Aamir Ali Syed, Shahid Khattak, Ali Raza Khan

**Affiliations:** Department of Surgical Oncology, Shaukat Khanum Memorial Cancer Hospital, House No. 164, Lane No. 7, J-3 Block Wapda Town Phase 1, Lahore 54000, Pakistan

## Abstract

*Background*. Two common procedures for esophageal resection are Ivor Lewis esophagectomy and transhiatal esophagectomy. Both procedures have high morbidity rates of 20–46%. Minimally invasive esophagectomy has been introduced to decrease morbidity. We report initial experience of MIE to determine the morbidity and mortality associated with this procedure during learning phase.* Material and Methods*. Patients undergoing MIE at our institute from January 2011 to May 2013 were reviewed. Record was kept for any morbidity and mortality. Descriptive statistics were presented as frequencies and continuous variables were presented as median. Survival analysis was performed using Kaplan Meier curves.* Results*. We performed 51 minimally invasive esophagectomies. Perioperative morbidity was in 16 (31.37%) patients. There were 3 (5.88%) anastomotic leaks. We encountered 1 respiratory complication. Reexploration was required in 3 (5.88%) patients. Median operative time was 375 minutes. Median hospital stay was 10 days. The most frequent long-term morbidity was anastomotic narrowing observed in 5 (9.88%) patients. There were no perioperative mortalities. Our mean overall survival was 37.66 months (95% confidence interval 33.75 to 41.56 months). Mean disease-free survival was 24.43 months (95% CI 21.26 to 27.60 months).* Conclusion*. Minimally invasive esophagectomy, when performed in the learning phase, has acceptable morbidity and mortality.

## 1. Introduction

Neoadjuvant treatment for locally advanced esophageal cancer is the standard of care [1, 2]. Esophagectomy has historically remained a very morbid procedure [3]. There are various modes of intervention among both open and minimally invasive groups. Previously, it was considered that transthoracic esophagectomy is the standard of care for oncological resection, but Orringer challenged this claim in the 1970s and redescribed the transhiatal procedure as equally effective but less morbid than transthoracic procedure owing to less postoperative pulmonary complications [4].

With the advent of laparoscopes, there has been a gradually increasing interest in minimally invasive procedures. Minimally invasive esophagectomy is a well-established intervention for esophageal resection. It is a complex procedure requiring greater operative time, but it is associated with shorter hospital stay and lesser blood transfusion requirements [5–8].

At our institution open esophagectomy remains the standard mode of intervention since 1998. Since 2011, we have started performing minimally invasive esophagectomy, with thoracic part of the procedure being performed by a thoracic surgeon. There are no studies from Pakistan reporting initial experience of morbidity and mortality for MIE. We decided to look into our experience of MIE to report morbidity and mortality with this procedure, during initial 51 cases. We hope that this experience will help other institutes from developing world to identify what to expect when starting with new MIE. We expect MIE to improve hospital stay and decrease morbidity and mortality.

This is a review of morbidity and mortality for first 51 MIE procedures for locally advanced esophageal cancer, that were resectable, on post neoadjuvant computerized tomography (CT) of chest and abdomen.

## 2. Material and Methods

All patients undergoing MIE at Shaukat khanum Memorial Cancer Hospital, for resectable esophageal cancers between January 2011 and May 2013, were reviewed. An approval was taken from the Ethical Review Committee. Data was prospectively collected. Patients were staged according to AJCC 7th edition classification for esophageal cancer. Pretreatment upper GI endoscopy, endoscopic ultrasound (EUS), and CT chest and abdomen with IV contrast were performed to clinically stage the disease. Patients were discussed in multidisciplinary team meetings. Patients with locally advanced disease (patients with T3 or above and/or N1 disease) require neoadjuvant chemoradiotherapy prior to surgical intervention. Patients received external beam radiation between 45 and 55 Gy with concurrent cisplatin and 5FU. Restaging scans were performed 4 weeks after the completion of XRT. Patients with resectable disease then underwent minimally invasive esophagectomy (MIE). All patients underwent elective surgeries.

VATS part of the procedure was performed by a thoracic surgeon, while abdominal and cervical part was performed by two surgeons. Record was kept for any intraoperative complication as well as postoperative wound infection, anastomotic leak, chyle leak, postoperative collections and any respiratory or cardiac problems requiring second intervention or prolonged stay in ICU and hospital. All complications and management provided for them were recorded. For delayed complications (occurring after 30 postoperative days), record was kept for significant aspiration causing repeated emergency room visits or admissions, anastomotic narrowing requiring dilatation or stent placement, and delayed gastric emptying requiring motility enhancing agents.

Patients were reviewed 10 days after discharge and then at the end of the first month and quarterly for 2 years. On each visit, patients undergo history and physical examination with any significant complaint of dysphagia to solids prompting endoscopy for evaluation of anastomosis and any suspicious area, biopsied. For patients with palpable cervical nodes or other significant clinical findings, a CT scan of that area would be required to exclude metastatic disease. In case of nonsignificant issues, a CT scan was repeated every year.

### 2.1. Surgical Procedure

Patients were placed in left lateral position and VATS was performed by a thoracic surgeon using 4 ports. Once esophageal mobilization was complete from thoracic inlet till hiatus, a chest drain was placed and all wounds were closed in layers. Patients were then changed to modified lithotomy position and abdominal part of the procedure was performed and stomach tube was created based on right gastric and right gastroepiploic vessels. Cervical part of the procedure was then performed through a hockey shaped incision along left sternocleidomastoid. Esophageal mobilization was performed in neck, saving the left recurrent laryngeal nerve. Esophagus was transected and specimen was delivered in abdomen. Using Alexis wound protector, specimen was retrieved through upper abdominal incision. PEG site was completely removed through this incision and specimen was delivered. Gastric tube was than fashioned using GIA 100. Tube was then passed through posterior mediastinum to neck, where single layer hand sewn esophagogastric anastomosis was performed using interrupted sutures of prolene 4/0. Suction drain was left in posterior mediastinum which was removed after 48 hours.

### 2.2. Statistical Analysis

Frequencies were calculated for descriptive statistics, while means were calculated for all continuous variables. Overall survival was calculated from date of diagnosis to date of death or last follow-up. Disease-free survival was calculated from date of surgery till date of recurrence. Kaplan Meier survival curves were used to determine overall and disease-free survival. Analysis was done using SPSS 19.

## 3. Results

During this period, we performed 51 minimally invasive esophagectomies. Clinical and pathological characteristics for patients were shown in Table 1. All patients were given neoadjuvant chemoradiotherapy prior to surgical intervention. 17/51 (33.33%) patients belong to stage II while 34/51 (66.66%) were stage III on initial staging workup. Two patients had stage T4b disease with significant radiological and clinical response to treatment. Both patients underwent minimally invasive esophagectomy. All patients had R0 resection, and 1 patient in minimally invasive group was found to have disease at PEG site on final histopathology. Perioperative morbidity was in 16 (31.37%) patients, as in Table 2. Wound infection occurred in 3 (5.88%) patients. Out of these, 2 patients had superficial wound infection of PEG site excision wound, while 1 had infection of VATS wound. None of the patients required debridement. There were 3 (5.88%) anastomotic leaks. All patients were managed conservatively with placement of nasojejunal tube for feeding, which was later removed once contrast study at three weeks showed no leak and patient started tolerating oral diet. We encountered 1 respiratory complication. Patient suffered from hospital acquired pneumonia. We encountered 2 chyle leaks, and both required thoracotomy and ligation of thoracic duct. Reexploration was required in 3 (5.88%) patients, 2 had chyle leak, while 1 had collection in mediastinum. Median operative time was 375 minutes. Median ICU stay was 1 day. Median hospital stay was 10 days, as in Table 3. Most frequent long-term morbidity was anastomotic narrowing observed in 5 (9.88%) patients. There were no perioperative mortalities.

**Figure 1:**
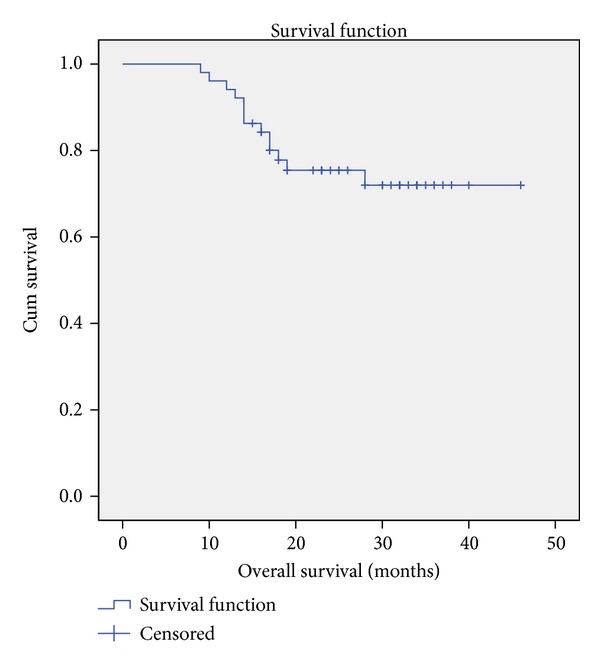
Overall survival.

**Figure 2:**
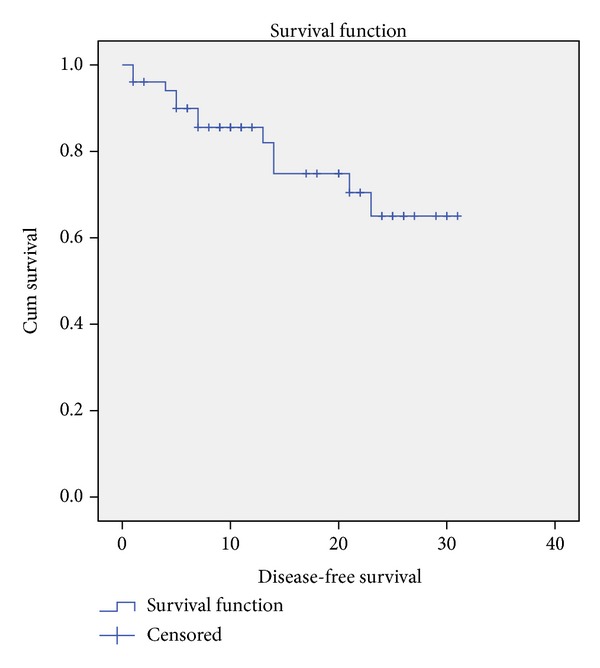
Disease-free survival.

Our median follow-up was 23 months. There were 13 (23.6%) deaths during this period. Our mean overall survival was 37.66 months (95% confidence interval 33.75 to 41.56 months), as in Figure 1. There were 12 (23.5%) recurrences during follow-up. Mean disease-free survival was 24.43 months (95% CI 21.26 to 27.60 months), as in Figure 2.

## 4. Discussion

Over the past decade many institutions had been performing minimally invasive esophagectomy as an alternative to open procedure and had shown it to be equally safe [9–12]. In our data, we had presented the outcomes of MIE. These were the first 51 MIE procedures being performed, so we had provided the perspective of how safe the procedure was when starting initially.

Schopmann et al. in his experience of 55 esophagectomies had described morbidity rate of 40% [13]. Zingg et al. in his study had shown morbidity rate of 34.5% [14]. In our experience 31.37% of patients undergoing MIE suffered some morbidity. Our MIE morbidity rates were comparable to international data.

Only 1 participant of our MIE group suffered hospital acquired pneumonia. In another study Schopmann et al. had shown pneumonia rates of 6.2% [15]. Literature reports pulmonary morbidity around 22%, but definition of major and minor respiratory complications were not well defined. Our better results were likely related to the consistent use of preoperative breathing exercises, postoperative thoracic epidural for pain control, and restarting early chest physiotherapy and incentive spirometry. All these measures had resulted in providing better results in terms of respiratory morbidity.

Three patients (5.88%) required thoracotomy, one for mediastinal collection drainage and two for thoracic duct ligation. International literature shows reintervention rate of 3–10%. However, Schopmann et al. had reintervention rate of 20%.

When looked at morbidity after 6 months, 11.6% patients suffered long-term morbidity. Major concern was anastomotic narrowing confirmed on postoperative endoscopy. 9.8% patients suffered this morbidity in our study.

All our patients had R0 resection and median lymph node yield was 11. Though we were expecting extra lymph node yield with MIE, during the initial phase of our study thoracic duct was not taken as part of specimen and also subcarinal lymph nodes were not taken en block with esophagus. However with incorporation of the aforementioned steps, our lymph node yield increased to over 20 lymph nodes per resection [16].

Results for our hospital stay and ICU stay have been skewed by 2 chyle leak patients as they both required longer hospital stay of 31 and 29 days. Also, their ICU stay was 21 days for the 1st patient and 16 days for the 2nd patient. Generally, all our esophagectomy patients stay in ICU for 24 hours after surgery. We get our contrast study on the 6th day and the patients go home on the 8th day [17].

Limitations include small sample size. However, this is the first data about outcomes of MIE from Pakistan and we hope that it will encourage other institutions in developing nations to adopt this technique safely.

## 5. Conclusion

Minimally invasive esophagectomy when performed in the learning phase has acceptable morbidity and mortality.

## Figures and Tables

**Table 1:** Patient characteristics.

	MIE	%
Age	51 years	21–71 years
Gender		
Male	23	45.09
Female	28	54.91
Comorbidity	7/51	13.73
BMI	20.91	13.93–32
Diagnosis		
Adenocarcinoma	10	19.6
Squamous cell carcinoma	41	80.4
Clinical T stage		
T1	0	
T2	6	11.8
T3	38	74.5
T4a	5	9.8
T4b	2	3.9
Clinical stage		
Stage I	0	
Stage II	17	33.33
Stage III	34	66.66
Stage IV	0	
Pathological stage		
CR	23	45.09
Stage I	2	3.92
Stage II	23	45.09
Stage III	3	5.88
Stage IV	0	

CR: Complete pathological response.

BMI: Body mass index.

**Table 2:** Morbidities for MIE.

Morbidity	MIE	%
Anastomotic leak	3/51	5.88
Respiratory complications	1/51	1.96
Arrhythmias	1/51	1.96
Postoperative hemorrhage	0/51	
Wound infection	3/51	5.88
Chyle leak	2/51	3.92
Collections	2/51	3.92
Reoperations	3/51	5.88
Anastomotic narrowing	5/51	9.80
Recurrent aspiration	1/51	1.96

**Table 3:** Continuous variables.

	Median	Mean	Range
Operative time (minutes)	375	360	214–506
Hospital stay (days)	10	12.88	8–46
ICU stay (days)	1	2.43	1–21
Number of nodes retrieved	11	12	2–29
